# Efficacy and safety of endoscopic submucosal dissection for rectal tumors with or without dentate line involvement: a propensity score matching analysis

**DOI:** 10.3389/fonc.2026.1924015

**Published:** 2026-09-03

**Authors:** Yan Hu, Qiang Zhang, Limei Gu, Jun Xiao, Qide Zhang, Huimin Guo, Min Chen, Juanjuan Huang, Ying Zhou, Tingsheng Ling

**Affiliations:** 1 Digestive Endoscopy Center, Affiliated Hospital of Nanjing University of Chinese Medicine, Nanjing, China; 2 Digestive Endoscopy Center, Jiangsu Province Hospital of Chinese Medicine, Nanjing, China; 3 Department of Gastroenterology, Nanjing Drum Tower Hospital, Nanjing, China

**Keywords:** dentate line, endoscopic submucosal dissection, postoperative complications, propensity score matching, rectal tumor

## Abstract

**Objective:**

To evaluate the technical feasibility, safety, and therapeutic outcomes of endoscopic submucosal dissection (ESD) for rectal tumors extending to the dentate line (RTDLs) compared with non-RTDLs using propensity score matching (PSM).

**Methods:**

We retrospectively reviewed 127 patients with rectal tumors treated with ESD at two tertiary centers from November 2012 to December 2023 (51 RTDLs, 76 non-RTDLs). A 1:1 PSM based on age, sex, and lesion diameter yielded 51 matched pairs (102 patients in total). Procedural parameters, pathological outcomes (classified as low-grade dysplasia (LGD), high-grade dysplasia (HGD), and adenocarcinoma), postoperative complications, and long-term oncological outcomes were systematically analyzed.

**Results:**

Following 1:1 PSM, baseline characteristics were well balanced between the non-RTDLs and RTDLs groups (P>0.05). En bloc resection was achieved in 100.0% of cases in both groups. R0 resection rates were comparable between the non-RTDLs and RTDLs groups (98.04% vs. 96.08%, P>0.999). However, the RTDLs group required a significantly longer mean ESD procedure duration (99.3 ± 72.8 min vs. 56.0 ± 52.9 min, P = 0.002) and demonstrated lower dissection speed (5.7 ± 6.5 min/cm^2^ vs. 3.1 ± 2.4 min/cm^2^, P = 0.010). Pathologically, precancerous neoplasms predominated in both groups, with HGD accounting for 47.1% in non-RTDLs and 51.0% in RTDLs; invasive adenocarcinoma accounted for 9.8% and 15.7%, respectively. Postoperative anal pain was significantly more frequent in the RTDLs group (25.6% vs. 5.4%, P = 0.025). Numerical increases in delayed bleeding (7.7% vs. 0.0%, P = 0.241) and rectal stenosis (17.9% vs. 2.7%, P = 0.056) were observed in the RTDLs group, without reaching statistical significance. No perforations, local recurrences, distant metastases, or disease-related deaths occurred during follow-up (P>0.999).

**Conclusion:**

ESD is a safe and effective organ-preserving treatment for rectal tumors extending to the dentate line, achieving excellent en bloc and R0 resection rates comparable to non-RTDLs. However, RTDLs present substantial technical complexity, resulting in longer procedure times, lower dissection efficiency, and increased risk of postoperative anal pain. Careful preoperative planning, endoscopist expertise, and close postoperative surveillance are essential when managing RTDLs.

## Introduction

Colorectal cancer (CRC) remains one of the most common malignancies worldwide and represents an important public health burden (1, 2). With advances in endoscopic technology, endoscopic submucosal dissection (ESD) has become an established minimally invasive treatment option for selected superficial colorectal neoplasms, allowing en bloc resection and accurate pathological evaluation (3).

However, the management of rectal tumors extending to the dentate line (RTDLs) remains technically challenging. The anorectal junction has unique anatomical characteristics, including a narrow operative field, limited endoscopic maneuverability, difficulty in identifying tissue planes, and abundant vascular and neural structures. These factors increase procedural complexity and may contribute to postoperative complications, particularly anal pain and functional discomfort.

Although several studies have demonstrated the feasibility and safety of ESD for anorectal junction lesions, evidence specifically focusing on RTDLs remains limited. Previous studies, including those by Imai et al. and Ferreira et al., reported that ESD could achieve favorable en bloc and R0 resection rates for lesions involving the dentate line, while also highlighting increased procedural difficulty and postoperative complications (4, 5). Therefore, the comparative clinical outcomes of ESD for RTDLs remain incompletely characterized.

Propensity score matching (PSM) is a statistical approach that reduces confounding effects in retrospective observational studies by balancing baseline characteristics between comparison groups. In this study, we performed a propensity score-matched analysis to compare the efficacy and safety of ESD between RTDLs and non-RTDLs lesions in a Chinese cohort over an 11-year period.

The aim of this study was to evaluate procedural characteristics, pathological outcomes, and postoperative complications associated with ESD for RTDLs and to provide additional clinical evidence regarding the management of these technically challenging lesions.

## Materials and methods

### Study subjects

A retrospective analysis was conducted on the clinical data of 127 patients with rectal tumors who received ESD treatment at the Digestive Endoscopy Center of Jiangsu Provincial Hospital of Chinese Medicine and the Department of Gastroenterology of Nanjing Drum Tower Hospital from November 2012 to December 2023.

Inclusion criteria: (1) age ≥ 18 years old; (2) definite diagnosis of rectal tumor and receipt of ESD treatment.

Exclusion criteria: (1) received other endoscopic treatments or open surgical resection; (2) incomplete clinical and follow-up data.

Among all enrolled patients, 51 cases were classified into the RTDLs group, and 76 cases into the non-RTDLs group. A 1:1 PSM was performed with a caliper value of 0.2 to match baseline variables, and 51 paired cases were finally included in each group for comparative analysis. This study was approved by the Ethics Committee of Jiangsu Provincial Hospital of Chinese Medicine, and informed consent was obtained from all patients and their families.

### Endoscopic equipment and procedure

All ESD procedures were performed by experienced endoscopists with extensive experience in colorectal ESD. All endoscopists had surpassed the learning curve for ESD and routinely performed ESD and EMR procedures during the study period. The main instruments included Olympus endoscopes (GIF-H290Z, GIF-Q260J, Olympus, Japan), dual knives (MK-T-1-195, Micro-Tech, China), transparent caps (D-201-10704, Olympus, Japan), endoscopic injection needles (NM-200U-0423, Olympus, Japan), electrocoagulation forceps (HBF-23/2000, Micro-Tech, China), high-frequency electrosurgical generator (VIO200D, ERBE, Germany), and endoscopic spray tubes (PW-5L-1, Olympus, Japan). The medications used were lidocaine injection, methylene blue injection, epinephrine, 0.2% ± 0.05% indigo carmine mucosal stain, and 0.75% compound iodine solution.

### Preoperative evaluation

All patients received comprehensive preoperative examinations, including routine blood tests, liver and renal function tests, coagulation function detection, tumor marker measurement, and abdominal computed tomography (CT) scanning. The indications for ESD were determined based on a comprehensive preoperative assessment, including biopsy pathology, magnifying endoscopy, narrow-band imaging (NBI), and radiological evaluation. ESD was considered for lesions with a high probability of complete en bloc resection and without evidence of lymph node or distant metastasis. For lesions suspected of adenocarcinoma based on biopsy results, ESD was performed when the lesion was considered suitable for endoscopic curative resection, including intramucosal carcinoma or superficial submucosal invasion without high-risk features. Magnifying endoscopy and NBI were used to evaluate surface and vascular patterns associated with invasion depth. Contrast-enhanced computed tomography (CT) was performed before treatment to assess lymph node involvement and distant metastasis. Lesions with suspected deep submucosal invasion, lymph node metastasis, or non-curative resection criteria were considered unsuitable for ESD and were referred for surgical treatment. Additionally, patients and their families were fully informed of the disease condition, surgical protocol, potential postoperative complications, and prognosis, and signed written informed consent before surgery.

### ESD procedure

ESD was performed using standard techniques according to institutional practice. The main procedural steps included:

1. Lesion boundary marking: For squamous epithelial lesions involving the dentate line, marking was performed 3–5 mm outside the lesion edge. For low rectal glandular epithelial lesions with clear boundaries, additional marking was not required.
2. Submucosal injection: Mixed solution containing normal saline, lidocaine, methylene blue and epinephrine was injected at multiple points outside the lesion edge to elevate the lesion and separate the mucosal layer from the muscular layer, facilitating subsequent dissection.
3. Marginal incision: A circular incision was made 3–5 mm outside the marking points, reaching the submucosal layer and close to the muscular layer. For lesions with a diameter exceeding 5 cm, the tunnel technique was adopted in part of cases, with only the anal-side mucosal edge incised.
4. Lesion dissection: The lesion tissue was completely dissected close to the proper muscular layer. Perforating blood vessels were carefully coagulated and hemostasized intraoperatively, and thorough hemostasis and wound cleaning were performed after complete lesion resection.
5. Pathological evaluation: The resected specimens were flattened and fixed on specimen plates, and stained with iodine solution or crystal violet again to confirm complete resection. All specimens were fixed in 10% neutral formalin for 24 hours, cut into tissue strips with a width of 2.0–3.0 mm, embedded in paraffin, sectioned and stained. The circumferential resection margin, basal resection margin, vascular tumor thrombus and nerve invasion were evaluated pathologically.

### Postoperative management

All patients were fasted for 24 hours postoperatively, and routine antibiotic prophylaxis was not administered. Intensive fluid replacement and nutritional support were provided. Postoperative anal canal drainage tube placement was selectively performed in patients considered at increased risk of local complications, including those with muscular layer injury, incomplete closure after muscle injury, or endoscopic full-thickness resection. The drainage tube was generally removed 36–48 hours after the procedure when the drainage fluid showed no evidence of abnormal bleeding and when the patient had no clinical symptoms suggestive of complications, including abdominal pain, fever, or perianal swelling. Patients with severe postoperative anal pain received symptomatic analgesic treatment. Postoperative complications including hematochezia and perineal subcutaneous emphysema were closely monitored during hospitalization.

### Postoperative follow-up

The first follow-up colonoscopy was performed at 3 months after surgery to evaluate wound healing. Subsequent colonoscopy reexaminations were conducted at 6 and 12 months postoperatively. For patients with pathologically confirmed superficial malignant tumors, abdominal CT scanning was additionally performed at 6 months and 1 year after surgery to assess tumor residual, recurrence and lymph node metastasis.

### Outcome measures

The primary outcomes were: ESD procedure duration; R0 resection rate; postoperative complications.

Secondary outcomes included: pathological characteristics; dissection efficiency; delayed bleeding; anal pain; rectal stenosis; recurrence; metastasis.

ESD procedure duration was defined as the interval from initial mucosal incision to completion of lesion removal and hemostasis.

Dissection efficiency was calculated as: Dissection speed= Procedure duration (min)/Lesion area (cm²).

### Statistical analysis

Statistical analyses were performed using SPSS version 26.0 (IBM Corp., Armonk, NY, USA). Continuous variables were expressed as mean ± standard deviation (SD) or median with interquartile range where appropriate. Categorical variables were presented as frequencies and percentages. For comparisons between groups, independent-sample t tests or nonparametric tests were used for continuous variables, while chi-square tests or Fisher’s exact tests were applied for categorical variables. A two-sided P value <0.05 was considered statistically significant.

## Results

### Baseline characteristics before and after PSM

A total of 127 patients with rectal tumors undergoing ESD were initially enrolled, comprising 76 patients in the non-RTDLs group and 51 patients in the RTDLs group. Before PSM, baseline parameters such as age, sex, ASA classification, circumferential extension, and antiplatelet/anticoagulant therapy were comparable between the two groups (P>0.05 for all). However, significant differences were observed in baseline lesion diameter (4.6 ± 3.0 cm vs. 7.0 ± 3.8 cm, P<0.001) and distance from the anal verge (5.3 ± 1.4 cm vs. 1.7 ± 1.3 cm, P<0.001).

After 1:1 nearest-neighbor PSM based on age, sex, and lesion diameter as matching variables with a caliper width of 0.2, 51 matched pairs (102 patients in total) were successfully generated. Following matching, lesion diameter showed no statistically significant difference between the non-RTDLs and RTDLs groups (6.0 ± 3.0 cm vs. 7.0 ± 3.8 cm, P = 0.136). All other clinical and demographic characteristics, including age (62.5 ± 11.2 vs. 61.3 ± 13.1 years, P = 0.622), male proportion (60.8% vs. 49.0%, P = 0.320), ASA classification I–II (90.2% vs. 86.3%, P = 0.759), circumferential extension ≥50% (21.6% vs. 29.4%, P = 0.495), antiplatelet therapy (9.8% vs. 11.8%, P> 0.999), and anticoagulant therapy (2.0% vs. 3.9%, P>0.999), were well balanced (P>0.05). The distance from the anal verge remained significantly shorter in the RTDLs group (1.7 ± 1.3 cm vs. 5.4 ± 1.5 cm, P<0.001), reflecting the intrinsic anatomical location defining lesions extending to the dentate line (**Table 1**).

**Table 1:** Comparison of baseline characteristics between the two groups before and after PSM(_x ± S).

Parameter	Before matching			After matching (1:1 PSM)		
	Non-RTDLs group (n=76)	RTDLs group (n=51)	*P*-value	Non-RTDLs group (n=51)	RTDLs group (n=51)	*P*-value
**Age** (years)	63.8 ± 10.7	61.3 ± 13.1	0.242	62.5± 11.2	61.3 ± 13.1	0.622
**Sex** [n (%)]			0.342			0.320
Male	45 (59.2%)	25 (49.0%)		31 (60.8%)	25 (49.0%)	
Female	31 (40.8%)	26 (51.0%)		20 (39.2%)	26 (51.0%)	
ASA classification [n(%)]			0.612			0.759
ASA I-II	69 (90.8%)	44 (86.3%)		46 (90.2%)	44 (86.3%)	
ASA III	7 (9.2%)	7 (13.7%)		5 (9.8%)	7 (13.7%)	
Lesion dia meter (cm)	4.6 ± 3.0	7.0 ± 3.8	< 0.001	6.0 ± 3.0	7.0 ± 3.8	0.136
Distance from anal verge (cm)	5.3 ± 1.4	1.7 ± 1.3	< 0.001	5.4 ± 1.5	1.7 ± 1.3	< 0.001
Circumferential extension [n (%)]			0.296			0.495
< 50%	61 (80.3%)	36 (70.6%)		40 (78.4%)	36 (70.6%)	
≥50%	15 (19.7%)	15 (29.4%)		11 (21.6%)	15 (29.4%)	
Antiplatelet therapy [n(%)]			0.867			> 0.999
Yes	7 (9.2%)	6 (11.8%)		5 (9.8%)	6 (11.8%)	
No	69 (90.8%)	45 (88.2%)		46 (90.2%)	45 (88.2%)	
Anticoagulant therapy [n(%)]			> 0.999			> 0.999
Yes	3 (3.9%)	2 (3.9%)		1 (2.0%)	2 (3.9%)	
No	73 (96.1%)	49 (96.1%)		50 (98.0%)	49 (96.1%)	

### Procedural and resection outcomes

The main ESD-related procedural outcomes after matching are summarized in **Table 2**. En bloc resection was successfully achieved in all 102 matched patients (100.0% vs. 100.0%, P>0.999). The R0 resection rates were similarly high between the non-RTDLs and RTDLs groups (98.04% vs. 96.08%, P > 0.999).

**Table 2:** The ESD-related outcomes after propensity score matching.

Outcome variable	Non-RTDLs group (n=51)	RTDLs group (n=51)	*P*-value
ESD procedure duration (min)	56.0 ± 52.9	99.3 ± 72.8	0.002
Dissection speed (min/cm²)	3.1 ± 2.4	5.7 ± 6.5	0.010
En bloc resection rate [n(%)]	51 (100.0%)	51 (100.0%)	>0.999
R0 resection rate [n(%)]	50 (98.04%)	49 (96.08%)	>0.999

However, performing ESD for lesions involving the dentate line was associated with higher procedural difficulty. Compared with the non-RTDLs group, the RTDLs group required a significantly longer mean procedure duration (99.3 ± 72.8 min vs. 56.0 ± 52.9 min, P = 0.002) and demonstrated a lower dissection speed (5.7 ± 6.5 min/cm² vs. 3.1 ± 2.4 min/cm², P = 0.010) (**Table 2**).

### Pathological characteristics

According to clinically relevant pathological categories, lesions in the non-RTDLs group consisted of low-grade dysplasia (LGD) in 22 cases (43.1%), high-grade dysplasia (HGD) in 24 cases (47.1%), and invasive adenocarcinoma in 5 cases (9.8%). In the RTDLs group, LGD, HGD, and adenocarcinoma accounted for 17 cases (33.3%), 26 cases (51.0%), and 8 cases (15.7%), respectively (**Table 3**). Positive lateral resection margins were observed in three patients. In the non-RTDLs group, one patient with a positive lateral margin underwent additional surgical resection. The surgical specimen showed high-grade intraepithelial neoplasia without definite vascular invasion, lymphatic metastasis, or perineural invasion, corresponding to pathological stage TisN0cM0. In the RTDLs group, two patients had small areas of low-grade intraepithelial neoplasia at the lateral resection margin. Because both lesions involved the anal margin and the patients were elderly, the potential benefits and risks of additional treatment were discussed in detail. After informed consent was obtained, close surveillance was selected. During follow-up, neither local recurrence nor distant metastasis was observed.

**Table 3:** Pathological characteristics according to clinically relevant categories[n(%)].

Pathological classification	Non-RTDLs group	RTDLs group
Low-Grade Dysplasia (LGD)	22 (43.1%)	17 (33.3%)
High-Grade Dysplasia (HGD)	24 (47.1%)	26 (51.0%)
Adenocarcinoma	5 (9.8%)	8 (15.7%)
Total	51	51

### Postoperative complications and oncological outcomes

Effective post-procedural follow-up data were obtained from 37 patients in the non-RTDLs group and 39 patients in the RTDLs group (**Table 4**).

**Table 4:** Postoperative complications and follow-up outcomes[n(%)].

Parameter	Non-RTDLs group (n=37)	RTDLs group (n=39)	P value
Complications			
Postoperative Anal Pain	2 (5.4%)	10 (25.6%)	0.025
Delayed Bleeding	0 (0.0%)	3 (7.7%)	0.241
Rectal Stenosis	1 (2.7%)	7 (17.9%)	0.056
Perforation	0 (0.0%)	0 (0.0%)	>0.999
Oncological Outcomes			
Local Recurrence	0 (0.0%)	0 (0.0%)	>0.999
Distant Metastasis	0 (0.0%)	0 (0.0%)	>0.999
Disease-Related Death	0 (0.0%)	0 (0.0%)	>0.999

Patients with complete follow-up data.

Regarding procedural safety endpoints, no intraoperative micro- or macro-perforations occurred in either group (0.0% vs. 0.0%, P>0.999). The incidence of postoperative anal pain was significantly higher in the RTDLs group compared to the non-RTDLs group (25.6% vs. 5.4%, P = 0.025). Most cases of postoperative anal pain were mild and relieved spontaneously or with routine symptomatic management. Patients with moderate-to-severe pain or persistent discomfort received additional analgesic treatment, including topical lidocaine or indomethacin suppositories when necessary. Although delayed bleeding and rectal stenosis occurred more frequently in the RTDLs group, these differences did not reach statistical significance (17.9% vs. 2.7%, P = 0.056). Symptomatic rectal stenosis was observed in a limited number of patients. These patients were treated with endoscopic incision of the stenotic segment combined with self-administered anal dilator therapy, resulting in improvement of symptoms during follow-up.

Regarding long-term oncological outcomes during surveillance, no local recurrence, distant metastasis, or disease-related deaths were detected in either group during the follow-up period (P>0.999 for all) (**Table 4**).

## Discussion

ESD has become an established organ-preserving treatment for selected superficial colorectal neoplasms because it enables en bloc resection, accurate histopathological assessment, and avoidance of radical surgery. However, RTDLs remain technically demanding because of the specific anatomical characteristics of the anorectal junction (6).

In this propensity score-matched retrospective study, we compared the clinical outcomes of ESD between RTDLs and non-RTDLs lesions. After adjustment for relevant baseline characteristics, RTDLs were associated with longer ESD procedure duration, lower dissection efficiency, and a higher incidence of postoperative anal pain. However, R0 resection rates were comparable between the two groups. These findings suggest that dentate line involvement mainly increases procedural complexity rather than preventing successful endoscopic treatment.

Several studies have evaluated the feasibility of ESD for rectal tumors involving the dentate line. Previous reports demonstrated that ESD can achieve favorable en bloc and R0 resection rates for these lesions, although technical difficulty and postoperative discomfort remain important considerations. A systematic review and meta-analysis by Zeng et al. showed that ESD for rectal tumors extending to the dentate line achieved acceptable efficacy and safety outcomes compared with non-involved lesions (7). More recent meta-analyses further supported the role of ESD as an effective treatment option for RTDLs while emphasizing the importance of appropriate patient selection and operator expertise (8, 9).

The present study contributes additional comparative evidence by applying propensity score adjustment in a relatively large real-world cohort from two experienced centers. Unlike previous case series that mainly described outcomes of RTDLs alone, our study directly compared RTDLs with non-RTDLs lesions after balancing important baseline factors. The findings provide further evidence regarding the specific procedural challenges associated with dentate line involvement.

The increased procedure duration and reduced dissection efficiency observed in the RTDLs group may be related to the technical characteristics of the anorectal junction. Lesions extending to the dentate line are located in a confined anatomical space, where endoscopic maneuverability is limited and maintaining an appropriate dissection plane can be challenging. These factors may prolong the procedure and require greater technical experience. Nevertheless, the comparable R0 resection rates between groups indicate that complete endoscopic resection can be achieved in appropriately selected patients.

Currently, several minimally invasive approaches are available for the management of distal rectal tumors, including ESD, transanal endoscopic microsurgery (TEM), transanal minimally invasive surgery (TAMIS), transanal endoscopic operation (TEO), and conventional transanal excision. These techniques have different technical principles, advantages, and limitations, and the optimal treatment strategy should be determined according to tumor characteristics, suspected depth of invasion, histological risk factors, and institutional expertise. TEM, introduced by Buess et al., is a transanal platform that enables precise full-thickness excision of rectal lesions under enhanced visualization and stable pneumorectum. It has been widely adopted for selected early rectal cancers and large adenomas that are unsuitable for conventional endoscopic resection (10). Compared with conventional transanal excision, TEM provides improved visualization, a larger working space, and more accurate excision margins, resulting in lower fragmentation rates and improved pathological assessment. However, TEM requires specialized equipment, dedicated training, and generally involves full-thickness rectal wall excision, which may increase the risk of postoperative defects, bleeding, or stenosis in selected cases (11). TAMIS was subsequently developed as a more accessible transanal minimally invasive platform by combining laparoscopic instruments with a transanal access port. Compared with TEM, TAMIS offers greater flexibility of available instruments and may facilitate the management of larger or more proximal rectal lesions (12). Similar to TEM, TAMIS allows full-thickness local excision and provides an intact specimen for pathological evaluation, making it a valuable option for lesions with suspected deeper submucosal invasion, non-lifting characteristics, or those considered unsuitable for endoscopic resection. However, because TAMIS and TEM involve excision beyond the submucosal layer, they may sacrifice normal rectal wall structures and are not primarily designed for purely mucosal or superficial submucosal lesions that can be completely removed by advanced endoscopic techniques (13). TEO represents another transanal endoscopic platform that shares similar principles with TEM, providing enhanced visualization and precise local excision through a transanal approach. Several studies have demonstrated comparable feasibility and local control outcomes between TEO and TEM for appropriately selected rectal lesions, although availability and operator experience vary among institutions (14, 15). Conventional transanal excision remains a technically simple and widely available approach; however, limitations including restricted visualization, difficulty in achieving precise margins, and higher risk of incomplete excision have restricted its application mainly to carefully selected distal rectal lesions (16).

In contrast, ESD enables en bloc removal of superficial rectal neoplasms while preserving the muscular layer and maintaining the anatomical integrity of the rectal wall. This organ-preserving characteristic is particularly relevant for lesions extending to the dentate line, where full-thickness excision may potentially increase the risk of postoperative functional impairment. Furthermore, ESD provides accurate histopathological evaluation of invasion depth and resection margins, which is essential for determining curative resection status (3). However, ESD for RTDLs remains technically challenging because of limited working space, difficult scope maneuverability, and the need to maintain an appropriate submucosal dissection plane close to the anal canal. Therefore, ESD and transanal surgical approaches should be considered complementary rather than competing strategies. TEM, TAMIS, and TEO may be preferable for lesions with suspected deeper invasion, lesions unsuitable for endoscopic resection, or cases requiring full-thickness excision. Conversely, ESD may be advantageous for superficial rectal tumors where complete en bloc resection can be achieved while preserving normal anatomy. For rectal tumors involving the dentate line, individualized treatment decisions based on tumor morphology, invasion risk, pathological characteristics, available expertise, and patient preference are essential to optimize both oncological safety and functional outcomes.

In our post-matching cohort, lesions in both groups were predominantly precancerous neoplasms, with high-grade dysplasia accounting for the largest proportion in the RTDLs group (51.0% vs. 47.1% in non-RTDLs). Although a slightly higher proportion of invasive adenocarcinomas was observed in lesions extending to the dentate line (15.7% vs. 9.8%), this numerical difference should be interpreted with caution. The substantial presence of HGD and invasive carcinomas in both arms underscores the importance of achieving high en bloc and R0 resection rates via ESD to enable precise histopathological staging and prevent localized disease progression. Because of the limited number of adenocarcinoma cases and relatively short follow-up duration, no definitive conclusions regarding long-term oncological outcomes can be drawn. Therefore, recurrence, metastasis, and disease-related mortality should be interpreted cautiously and considered exploratory outcomes requiring further validation.

Postoperative complications represent an important consideration when evaluating ESD for anorectal lesions. In our study, postoperative anal pain was significantly more common in patients with RTDLs (25.6% vs 5.4%). This finding is consistent with previous reports. Postoperative anal pain may be related to lesion location involving the dentate line, where abundant sensory nerve fibers are present, although the exact mechanism remains multifactorial. Although postoperative anal pain was significantly more frequent among patients with RTDLs, it was generally a transient symptom and rarely required invasive intervention. However, clinicians should be aware of the possibility of moderate-to-severe pain, particularly for lesions involving the dentate line. In our clinical practice, patients with persistent or clinically significant pain can be managed with additional topical analgesic therapy, such as lidocaine or indomethacin suppositories. In contrast to postoperative pain, rectal stenosis represents a clinically meaningful delayed complication because it may affect bowel function and require additional intervention. Fibrotic healing after extensive mucosal defects, especially in lesions with larger resection areas or circumferential involvement, may contribute to stenosis development. In our cohort, affected patients improved after endoscopic incision combined with anal dilator therapy. Although delayed bleeding and rectal stenosis occurred more frequently in the RTDLs group, statistical significance was not reached. The observed trends may reflect the technical complexity of lesions involving the dentate line; however, these findings require confirmation in larger prospective studies. Therefore, our results should be interpreted as hypothesis-generating rather than definitive evidence of increased complication risk.

The clinical contribution of this study is primarily based on its comparative design and methodological approach. Previous studies have mainly consisted of retrospective case series describing outcomes of anorectal junction ESD. By applying propensity score matching, our study attempted to reduce baseline imbalance and provide a more reliable comparison between RTDLs and non-RTDLs lesions. In addition, the inclusion of patients treated over an 11-year period from two experienced Chinese centers provides further real-world evidence regarding the feasibility of ESD in this challenging anatomical location.

Several limitations should be acknowledged. First, although propensity score matching improved baseline comparability between groups, this approach inevitably resulted in the exclusion of unmatched patients and may have reduced statistical power. To minimize this limitation, multivariable regression analyses using the cohort were performed as sensitivity analyses, and the results were consistent with the primary analysis. Second, the sample size remained relatively limited, particularly after matching, which may have reduced the statistical power to detect differences in uncommon complications such as delayed bleeding and stenosis. Third, follow-up duration was insufficient to draw definitive conclusions regarding long-term oncological outcomes. Finally, all procedures were performed at experienced centers, and therefore the results may not be directly generalizable to institutions with less experience in colorectal ESD. Future multicenter prospective studies with larger sample sizes are needed to further evaluate the optimal treatment strategy for RTDLs and to compare ESD with alternative approaches such as transanal minimally invasive surgery.

In conclusion, ESD is a feasible and effective minimally invasive treatment option for selected rectal tumors with or without dentate line involvement. After propensity score matching, RTDLs were associated with longer procedure duration and lower dissection efficiency, reflecting the technical challenges of this anatomical region. However, comparable R0 resection rates indicate that satisfactory treatment outcomes can be achieved by experienced endoscopists. Patients with RTDLs experienced significantly more postoperative anal pain. Therefore, careful patient selection, adequate preoperative counseling, and individualized postoperative management are recommended for patients undergoing ESD for RTDLs.

## Data Availability

The raw data supporting the conclusions of this article will be made available by the authors, without undue reservation.
