# Supplementary material for: Efficacy and safety of endoscopic submucosal dissection for rectal tumors with or without dentate line involvement: a propensity score matching analysis
Source: Front Oncol. 2026 Sep 3;16:1924015. doi: 10.3389/fonc.2026.1924015 (PMC13581859; doi:10.3389/fonc.2026.1924015)
Supplement: Supplementary file 1 [file Table1.docx]

**Sensitivity Analysis via Logistic Regression**

To complement the PSM strategy, we performed a multivariable logistic regression analysis incorporating key baseline characteristics and procedural metrics (Supplementary Table S1). Baseline demographics and pre-procedural factors, including age (OR 1.00, 95% CI 0.97-1.03, P=0.899), sex (OR 1.49, 95% CI 0.68-3.25, P=0.321), ASA score (OR 1.46, 95% CI 0.43-4.96, P=0.540), lesion diameter (adjusted OR 1.11, 95% CI 0.95-1.28, P=0.190), and antithrombotic therapy, were not significantly associated with dentate line status, confirming excellent inter-group comparability after matching. Crucially, multivariable regression demonstrated that ESD performed for RTDLs was independently associated with a significantly longer procedure duration (adjusted OR 1.01, 95% CI 1.00-1.02, P=0.010) and lower dissection speed (adjusted OR 1.23, 95% CI 1.05-1.43, P=0.010). These findings are in complete agreement with the primary PSM outcomes, confirming the technical difficulty of RTDL resection and the statistical reliability of our overall conclusions.

**Supplementary Table S1 Univariable and Multivariable Logistic Regression Analysis Evaluating Variable (N=102)**

| **Variable** |  | **Non-RTDLs Group (n=51)** | **RTDLs Group (n=51)** | **Univariable OR (95% CI)** | **P value** | **Multivariable OR (95% CI)** | **P value** |
| --- | --- | --- | --- | --- | --- | --- | --- |
| **Sex** | Female vs. Male | 20 (39.2%) vs. 31 (60.8%) | 26 (51.0%) vs. 25 (49.0%) | 1.49 (0.68-3.25) | 0.321 | - |  |
| **Age** | Years  (Mean±SD) | 62.5±11.2 | 61.3±13.1 | 1.00 (0.97-1.03) | 0.899 | - |  |
| **ASA Classification** | III vs. I-II | 5 (9.8%) vs. 46 (90.2%) | 7 (13.7%) vs. 44 (86.3%) | 1.46 (0.43-4.96) | 0.540 | - |  |
| **Lesion Diameter** | Cm  (Mean±SD) | 6.0±3.0 | 7.0±3.8 | 1.09 (0.97-1.23) | 0.140 | 1.11 (0.95-1.28) | 0.190 |
| **Distance from Anal Verge** | cm  (Mean±SD) | 5.4±1.5 | 1.7±1.3 | 0.00 (0.00-Inf) | 0.998 | - |  |
| **Circumferential Extension** | ≥50% vs. < 50% | 11 (21.6%) vs. 40 (78.4%) | 15 (29.4%) vs. 36 (70.6%) | 1.52 (0.62-3.72) | 0.365 | - |  |
| **Antiplatelet Therapy** | No vs. Yes | 46 (90.2%) vs. 5 (9.8%) | 45 (88.2%) vs. 6 (11.8%) | 0.82 (0.23-2.86) | 0.750 | - |  |
| **Anticoagulant Therapy** | No vs. Yes | 50 (98.0%) vs. 1 (2.0%) | 49 (96.1%) vs. 2 (3.9%) | 0.49 (0.04-5.58) | 0.566 | - |  |
| **ESD Procedure Duration** | min  (Mean±SD) | 56.0±52.9 | 99.3±72.8 | 1.01 (1.00-1.02) | 0.003 | 1.01 (1.00-1.02) | 0.010 |
| **Dissection Speed** | min/cm^2^  (Mean±SD) | 3.1±2.4 | 5.7±6.5 | 1.17 (1.02-1.34) | 0.029 | 1.23 (1.05-1.43) | 0.010 |
